# Global trends and emerging insights in ocular tumor-associated metabolites: a bibliometric and visualization analysis

**DOI:** 10.3389/fmolb.2025.1572710

**Published:** 2025-06-18

**Authors:** Subo Cai, Wuping Xu, Dongyi Yu, Yimin Xia

**Affiliations:** ^1^ Department of Ophthalmology, The Affiliated Jiangyin Hospital of Nantong University, Jiangyin, Jiangsu, China; ^2^ Department of Ophthalmology, The Affiliated Kunshan Hospital of Jiangsu University, Kunshan, Jiangsu, China; ^3^ Department of Ophthalmology, Yueyang Hospital of Integrated Traditional Chinese and Western Medicine Affiliated to Shanghai University of Traditional Chinese Medicine, Shanghai, China

**Keywords:** ocular tumors, metabolites, bibliometrics, visualization analysis, uveal melanoma

## Abstract

**Objective:**

This study aims to perform a comprehensive bibliometric analysis of global research on ocular tumor metabolomics, identifying key research trends, influential contributors, and emerging themes from 1995 to 2024.

**Methods:**

A systematic search was conducted in the Web of Science Core Collection (WoSCC) database to retrieve publications related to ocular tumor metabolomics from 1 January 1995, to 12 December 2024. Bibliometric analyses, including publication trends, citation networks, and keyword co-occurrence patterns, were performed using VOSviewer and CiteSpace. Collaborative networks, co-cited references, and keyword burst analyses were mapped to uncover shifts in research focus and global cooperation.

**Results:**

Both annual publication volume and citation frequency have shown a wave-like increase since 2000. Initially, research activity was limited during the early period (2000-2006), followed by a phase of slow growth between 2007 and 2015. A surge in publications occurred after 2016, peaking in 2022 with 81 articles. Despite the increase in publications, citation frequency declined after 2019, indicating a gap between research output and scholarly impact. The United States, China, and Italy were the top contributing countries, with the U.S. maintaining a leadership role. Keyword analysis revealed key research themes, including biomarkers, uveal melanoma, and immunotherapy, with a shift towards gene expression, tumor metastasis, and liquid biopsy in recent years. Keyword burst analysis identified retinoblastoma protein and gene expression as major research hotspots.

**Conclusion:**

Ocular tumor metabolomics research has made significant strides in recent years. This analysis provides a comprehensive framework for future research, emphasizing the need for interdisciplinary collaborations and the development of non-invasive diagnostic tools to improve the diagnosis, prognosis, and treatment of ocular tumors.

## Introduction

Ocular tumors are a heterogeneous group of neoplasms that predominantly affect the structures of the eye, including the uveal tract, retina, optic nerve, and surrounding tissues (Rene, 2013; Moon et al., 2021). These tumors can be broadly classified into intraocular malignancies, such as uveal melanoma and retinoblastoma, and extraocular malignancies, including eyelid cancers and lacrimal gland tumors (Banou et al., 2023; Dekmezian et al., 2013; Nagarkatti-Gude et al., 2012; Chan et al., 2024). Uveal melanoma is the most common primary intraocular malignancy in adults, arising primarily from melanocytes in the uveal tract (comprising the iris, ciliary body, and choroid) (Chattopadhyay et al., 2016; Spagnolo et al., 2012). While its incidence is relatively low, approximately 5–6 cases per million people annually, uveal melanoma is the leading cause of ocular malignancy (Rantala et al., 2022; Carvajal et al., 2017). It is known for its high metastatic potential, particularly to the liver, with studies indicating that 30%–50% of patients with uveal melanoma develop metastases (Carvajal et al., 2023). Despite advances in diagnosis and treatment, metastatic uveal melanoma remains associated with a poor prognosis, with a 5-year survival rate of less than 20% (Jager et al., 2020; Gelmi and Jager, 2024). On the other hand, retinoblastoma, a primary malignancy of the retina, is the most common intraocular tumor in children, with an incidence of approximately 1 in 15,000 live births, translating to an estimated 8,000–10,000 new cases globally each year (Villegas et al., 2013; Zhou et al., 2024). While survival rates have improved dramatically, reaching over 90% in high-income countries, retinoblastoma continues to cause significant childhood blindness in low- and middle-income regions due to challenges in early detection and access to care (Benavente and Dyer, 2015; de Jong et al., 2014). The disease is commonly associated with mutations in the RB1 gene, leading to uncontrolled proliferation of retinal cells (Farhat et al., 2022). Extraocular tumors, including eyelid malignancies such as basal cell carcinoma and squamous cell carcinoma, as well as lacrimal gland tumors, though less common, can also lead to severe visual impairment and require immediate clinical intervention (Lebrun et al., 2024). Eyelid cancers, which often present as skin lesions, are among the most common extraocular malignancies (Schoelles and Auw-Haedrich, 2024), while lacrimal gland tumors, though rarer, have been increasing in incidence in recent years (Gündüz et al., 2018).

Over the past 2 decades, significant advances have been made in understanding the molecular mechanisms driving ocular tumorigenesis (Jiang et al., 2020; Peters et al., 2022). While genetic mutations, particularly those involving tumor suppressor genes and oncogenes, have long been recognized as critical drivers of ocular tumors, emerging evidence suggests that factors such as the tumor microenvironment, immune evasion mechanisms, and metabolic reprogramming play pivotal roles in tumor development and progression (Liu et al., 2024; Xu et al., 2021; Xia L. et al., 2021). Metabolic reprogramming refers to the ability of tumor cells to alter their energy production and biosynthetic pathways—such as glycolysis, oxidative phosphorylation, and lipid metabolism—to support rapid proliferation, survival, and adaptation to the tumor microenvironment. This process is considered one of the hallmarks of cancer (Faubert et al., 2020). It involves the upregulation of key metabolic pathways, including glycolysis, oxidative phosphorylation, and lipid metabolism, which support enhanced biosynthesis and cellular energy production (Xia Y. et al., 2021; Yang et al., 2023). In ocular tumors, such metabolic shifts have been shown to correlate with aggressive tumor behaviors, such as increased proliferation, invasion, and metastasis (Jin et al., 2023; Lin et al., 2024). For instance, uveal melanoma cells often exhibit upregulation of glycolytic enzymes, enabling them to thrive in hypoxic conditions typical of the tumor microenvironment (Longhitano et al., 2022). Similarly, in retinoblastoma, altered lipid metabolism and dysregulated amino acid metabolism have been implicated in the modulation of cell cycle progression and apoptosis resistance (Zuo et al., 2023).

Recent research has focused on characterizing the metabolic landscape of ocular tumors by identifying key metabolites and metabolic pathways that may serve as biomarkers for early detection or therapeutic targets (Huang et al., 2020; Issaq and Heske, 2020). The growing recognition of the role of metabolic alterations in ocular tumors underscores the importance of exploring tumor-associated metabolites and metabolic networks (Pederson et al., 2023). These studies are not only crucial for understanding the pathophysiology of ocular malignancies but also for developing new strategies for diagnosis, prognosis, and treatment. Therefore, examining the intricate relationship between ocular tumors and their metabolic profiles has become an essential area of investigation, promising to reveal novel insights into ocular tumor biology and potential therapeutic avenues.

Despite significant advances in elucidating the molecular mechanisms underlying ocular tumor-associated metabolites, a critical gap remains in the quantitative analysis of research trends and collaborative dynamics within ocular tumor metabolomics. There is currently no comprehensive bibliometric assessment that maps the evolution of research themes or identifies the leading research institutions in the field. The lack of a systematic evaluation of the global research landscape constrains our ability to identify emerging areas of focus and to forecast future trends in this domain. In particular, bibliometric analysis provides a unique opportunity to identify underexplored research areas, objectively assess scholarly influence, and uncover thematic shifts over time—thus offering strategic guidance for future investigations. This gap emphasizes the necessity for rigorous bibliometric studies that can provide an in-depth understanding of the current state of ocular tumor metabolomics, pinpoint knowledge gaps, and offer valuable insights to guide the development of future therapeutic strategies.

This study aims to conduct a comprehensive bibliometric analysis to summarize the progress of the research on ocular tumor-associated metabolites, focusing on emerging trends in metabolic reprogramming. Through quantitative analysis of research topics, citation patterns, and collaborative networks, we aim to identify key areas in ocular tumor metabolomics, highlight leading institutions and authors, and provide a clearer view of the research landscape. By synthesizing existing knowledge and identifying research gaps, this study will offer valuable insights for future directions, helping to pinpoint emerging therapeutic targets and inform novel diagnostic and treatment strategies for ocular tumors.

## Materials and methods

### Data source and search strategy

This study utilized the Web of Science Core Collection (WoSCC) as the sole data source, given its recognized reliability and high accuracy in classifying and indexing scientific literature. To ensure comprehensive coverage of relevant publications, a well-defined search strategy was employed using the following query:TS=(“ophthalmic oncology” OR “ocular oncology” OR “eye tumors” OR “retinoblastoma” OR “uveal melanoma” OR “intraocular lymphoma” OR “ocular neoplasms” OR “ocular malignancies” OR “eye cancer”) AND TS=(“metabolomics” OR “metabolites” OR “metabolic profiling” OR “biomarkers” OR “metabolic pathways” OR “metabolic markers”).

The search spanned the database inception to 13 December 2024, and initially retrieved 727 records. Articles and reviews written in English were selected for analysis, while other publication types and languages were excluded. After exporting the records in “plain text” format with “full records and cited references,” a total of 677 publications were included for further analysis.

### Inclusion and exclusion criteria

The selection of publications for this study was conducted based on the following predefined inclusion criteria: (1) Full-text articles explicitly investigating ocular tumor-associated metabolites or metabolomic mechanisms in ocular oncology; (2) Peer-reviewed original research articles or review papers published in the English language; (3) Publications indexed in the Web of Science Core Collection (WoSCC) from inception through 13 December 2024.

Exclusion criteria encompassed: 1) Studies not directly related to ocular tumor metabolomics; (2) Non-academic or non-peer-reviewed documents, including conference proceedings, book chapters, corrections, editorials, and news items.

Although bibliometric data may be retrieved from other sources such as Scopus or PubMed, the WoSCC database was selected due to its high-quality citation indexing, standardized metadata structure, and compatibility with advanced bibliometric software tools. This ensures consistency and reliability in cross-sectional and longitudinal bibliometric analyses.

### Data categorization and preprocessing

To systematically organize and preprocess the collected publications, bibliometric parameters such as publication year, authorship, country or region, institutional affiliation, journal name, and citation counts were classified using Microsoft Excel. This structured dataset served as the foundation for identifying research patterns and trends.

The exported bibliographic data were uniformly named as “download” and imported into CiteSpace (version 6.2.R4) for format conversion and initial processing. The time span was set from 2000 to 2024, with a slice of 1 year. Term sources included titles, abstracts, and keywords, while the threshold for each time slice was set to the top 50 terms (Top N = 50), with k set to 15.

In VOSviewer, bibliometric mapping was initiated by selecting the “create a map based on bibliographic data” option. Terms were extracted from the “title and abstract fields,” enabling the construction of co-occurrence networks and clustering analyses. Additionally, Bibliometrix, a bibliometric R package, was used to import and analyze the data, facilitating comprehensive visualizations of collaboration networks, citation relationships, and dynamic trends in keywords. This combined approach ensured systematic data preprocessing and robust preparation for subsequent analyses.

### Data analysis and visualization

This study employed a combination of bibliometric tools to analyze and visualize the data comprehensively. CiteSpace was used to generate knowledge maps, including co-occurrence networks, keyword clustering, and temporal evolution timelines, providing insights into research hotspots and trends within the field. Burst detection functionality in CiteSpace was applied to identify keywords and references with significant short-term increases in research activity, highlighting pivotal shifts in the research landscape.

VOSviewer facilitated the construction of bibliometric maps to visualize co-authorship, co-citation, and keyword co-occurrence networks. These maps, with node sizes representing the frequency of publications or co-occurrences and colors differentiating thematic clusters, provided a clear depiction of research communities and collaboration structures.

Bibliometrix, implemented in the R programming environment, complemented the analysis by offering advanced bibliometric functionalities. These included author collaboration networks, citation impact metrics such as the h-index, and keyword trend analyses. By integrating the capabilities of these tools, this study delivered a comprehensive overview of the intellectual structure, collaborative networks, and evolving research trends in the field of metabolomics related to ophthalmic oncology.

### Ethical considerations

This study exclusively utilized publicly available data from the Web of Science Core Collection (WoSCC) database. As no human or animal subjects were involved, ethical approval was not required. All analyses were conducted in accordance with established bibliometric research practices, ensuring compliance with academic integrity and ethical standards.

## Results

### Analysis of annual publication volume and citation trends

**FIGURE 1:**
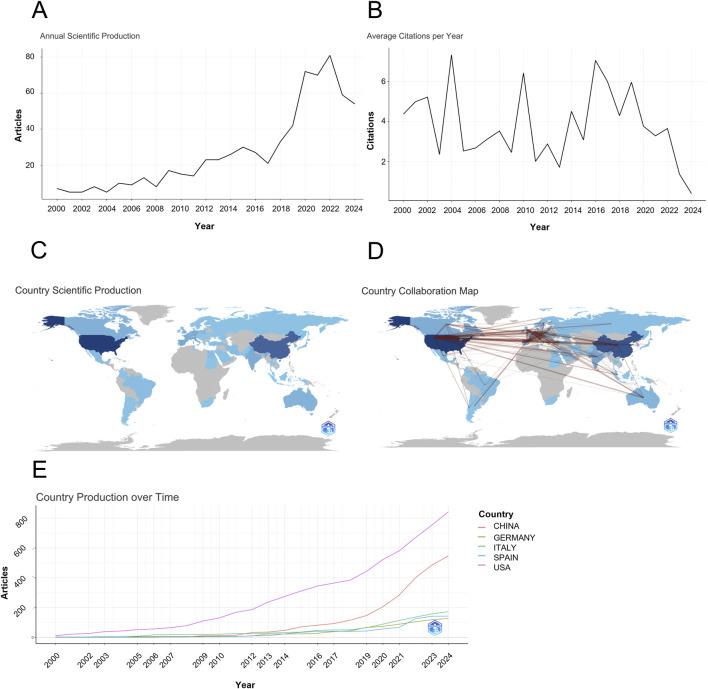
Trends in Ocular Tumor-Associated Metabolite Research. **(A)** Annual publication volume from 2000 to 2024. **(B)** Citation trends from 2000 to 2024. **(C)** Geographical distribution of international collaboration in ocular tumor research, highlighting the United States, China, and Italy as key contributors. **(D)** Network of countries involved in ocular tumor metabolite research. **(E)** Publication trend of top contributing countries over the last 2 decades.

To evaluate the progress of research on ocular tumor-associated metabolites, we analyzed the annual publication volume and citation trends (Figures 1A,B). Since the year 2000, the overall number of publications and citations has followed a wave-like upward trajectory, reflecting an increasing global interest in this area of research.

From 2000 to 2006, the field was in its nascent stage, with fewer than 10 articles published annually, and contributions from researchers were relatively sparse. This period marked the initial exploration of ocular tumor-related metabolites. Between 2007 and 2015, publication activity showed only a slow increase, with minimal growth in the volume of research. This stagnation phase suggests that while research was ongoing, it lacked significant breakthroughs or momentum.

A significant shift occurred after 2016, with a noticeable surge in publications. The peak in 2022, with 81 articles published, represents the most productive year in this field to date. However, despite the rising number of publications, citation frequency exhibited an unstable trend, with a decline observed from 2019 onward. This discrepancy suggests that while the volume of published research has increased, its impact and influence within the academic community have not kept pace. The decline in citations indicates that, although the field has reached new heights in terms of research output, its authority and recognition remain limited, and further efforts are needed to enhance its scholarly impact.

### National collaboration analysis

The geographical visualization of international collaborations in ocular tumor-related metabolite research is depicted in Figures 1C,D. The United States leads the global publication output in this field, with its first publication appearing as early as 2000, establishing its position as a pioneer in ocular tumor-related metabolite research. China and Italy follow, ranking second and third in publication volume, respectively. Notably, China entered this research domain later, with its earliest publication in 2009.

Although China’s involvement in this area began relatively late, the country has rapidly advanced, driven by substantial progress in its national medical research infrastructure. This has resulted in a dramatic increase in the number of publications, propelling China to the second position globally in terms of total publication output. Overall, 62 countries have contributed to the body of research in this domain, with 17 nations producing at least 10 publications, accounting for 27.4% of the total output. As shown in Table 1, the top three contributing countries are the United States (n = 227), China (n = 175), and Italy (n = 49).

**TABLE 1:** Country-wise contribution to ocular tumor-related metabolite research.

Index	Number of publications	Centrality	Publication year	Country of publication
1	227	0.08	2000	United States
2	175	0	2009	CHINA
3	49	0.26	2000	ITALY
4	48	0	2002	ENGLAND
5	38	0.08	2005	GERMANY
6	37	0.06	2007	INDIA
7	34	0.08	2007	SPAIN
8	30	0.26	2002	FRANCE
9	29	0.24	2001	CANADA
10	22	0.15	2005	NETHERLANDS

Analysis of publication trends using Bibliometrix over the period from 2000 to 2024 reveals that the United States has consistently maintained its leadership in global publication output. Over the past decade, China has firmly secured the second position, underscoring the significant contributions made by both countries to the advancement of ocular tumor-related metabolite research (Figure 1E).

### Institutional Co-occurrence analysis

**FIGURE 2:**
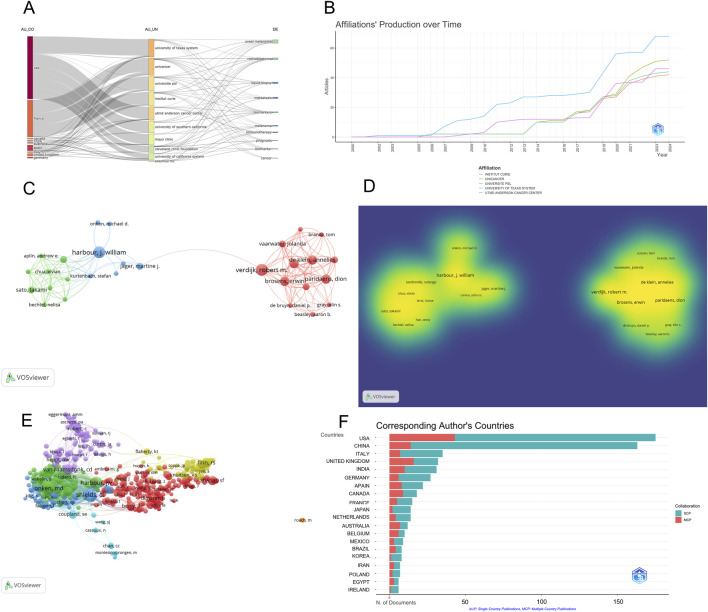
Institutional and Author Collaboration Networks. **(A)** Tree-field plot illustrating collaboration networks in ocular tumor-related metabolite research. **(B)** Temporal trends in publication output by leading institutions in ocular tumor-related metabolite research. **(C)** Author collaboration network map based on co-authorship, visualized with VOSviewer. The size of nodes represents the number of publications, with key contributors like Shields CL and Harbour JW identified. **(D)** Density visualization of author collaborations, showcasing the collaboration intensity among researchers. **(E)** Citation-based author collaboration network, highlighting prominent authors and their citation relationships. **(F)** Breakdown of authors by nationality, illustrating the prominence of U.S. and Chinese researchers.

The top ten institutions contributing to the literature in the field of ocular tumor-related metabolites are presented in Table 2. A visual analysis of the institutions publishing in this area reveals that six of the top ten institutions are based in the United States (University of Texas System, University of California System, Harvard University, UTMD Anderson Cancer Center), while two institutions are from China (Capital Medical University, Shanghai Jiao Tong University), and two are from France (Institut National de la Santé et de la Recherche Médicale [Inserm], UNICANCER). This distribution highlights the dominant academic influence of U.S. research institutions in the field of ocular tumor-related metabolites. However, a closer inspection of the overall institutional centrality shows relatively low values (Figures 2A,B). This indicates a need for greater international collaboration and the establishment of more robust research networks. Enhancing cooperative exchanges between institutions could foster significant advancements in ocular tumor-related metabolite research, ultimately leading to breakthrough discoveries in the field.

**TABLE 2:** Top 10 institutions by publication output in ocular tumor-related metabolite research.

Index	Number of publications	Centrality	Year	Publishing institution
1	22	0.11	2002	University of Texas System
2	16	0.04	2009	University of California System
3	15	0.18	2001	Harvard University
4	15	0.05	2014	Capital Medical University
5	14	0.05	2008	UTMD Anderson Cancer Center
6	12	0.03	2002	Institut National de la Sante et de la Recherche Medicale (Inserm)
7	12	0.02	2006	UNICANCER
8	11	0.03	2009	Cleveland Clinic Foundation
9	11	0.02	2000	University System of Ohio
10	11	0.01	2016	Shanghai Jiao Tong University

### Author collaboration visualization analysis

A visualization of author collaboration networks in the field of ocular tumor-related metabolites was generated using VOSviewer software. Authors with at least two publications were included, forming a collaboration network and density visualization map (Figures 2C,D). The size of the nodes and font in the network is proportional to the publication volume of each author. Among the included English-language papers, 311 authors were identified with a publication frequency of two or more papers. This network illustrates the collaboration between key contributors in the field.

An analysis of the author nationality distribution reveals that researchers from the United States and China significantly outnumber those from other countries, highlighting the global prominence and authority of research in this area, particularly from China. Prominent researchers, such as Shields CL and Harbour JW, represent the leading bodies of work in ocular tumor-related metabolite research (Table 3). Figures 2E,F present the author citation collaboration network and a breakdown of authors and their respective countries.

**TABLE 3:** Leading authors in ocular tumor-related metabolite research based on publication frequency.

Index	Publication frequency	Author name	Citation frequency	Author name
1	9	berry, jesse l	182	shields, cl
2	9	coupland, sarah e	143	harbour, jw
3	9	xu, liya	133	onken, md
4	8	peng, chen-ching	113	singh, ad
5	7	khetan, vikas	100	van raamsdonk, cd
6	7	singh, arun d	88	damato, b
7	6	kalirai, helen	82	finn, rs
8	6	krishnakumar, subramanian	72	kaliki, s
9	6	lotan, yair	69	shariat, sf
10	5	caltabiano, rosario	67	carvajal, rd

### Keyword visualization and Co-occurrence analysis

**FIGURE 3:**
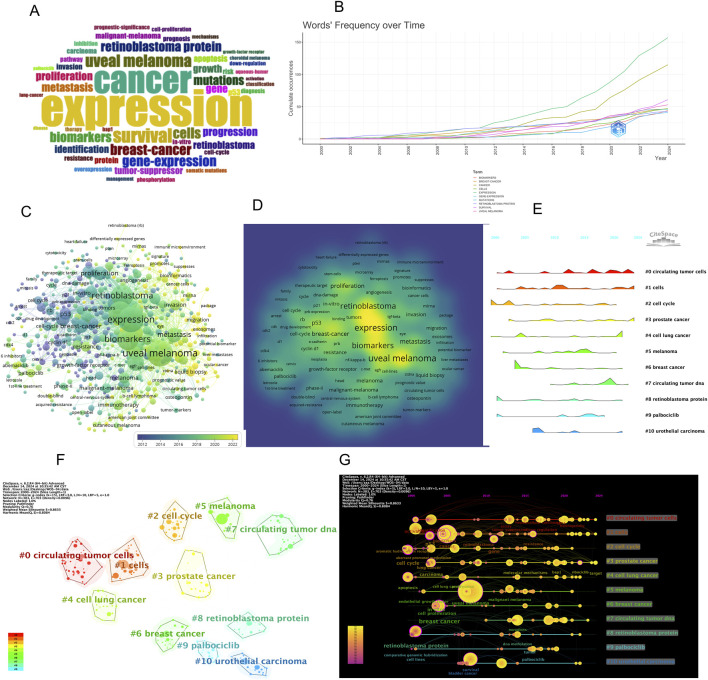
Keyword analysis and research trends in ocular tumor metabolite studies. **(A)** Word cloud of keywords in ocular tumor-related metabolite research. **(B)** Trends in keyword frequency over time. **(C)** Co-occurrence network of keywords in ocular tumor-related metabolite research. **(D)** keyword Co-occurrence density visualization in ocular tumor-related metabolite research. **(E)** cluster peak diagram of keyword themes over time. **(F)** Keyword clustering map for ocular tumor-related metabolite research. **(G)** timeline view of keyword clusters in ocular tumor metabolite research.

Keywords provide a condensed summary of the research topics in a given field. In this study, keywords were extracted from publications on ocular tumor-related metabolites indexed in the Web of Science (WOS) database. These keywords were analyzed through both a word cloud and a co-occurrence network map, offering a visual overview of the central themes in this area of research (Figures 3A–D).

The word cloud (Figure 3A) illustrates the prominence of each keyword based on its frequency of occurrence, with the largest terms representing those most frequently mentioned. The top 15 keywords identified through co-occurrence analysis include uveal melanoma, expression, cancer, retinoblastoma, biomarkers, prognosis, survival, metastasis, proliferation, breast cancer, cells, retinoblastoma protein, gene expression, mutations, and progression (Table 4). Among these, uveal melanoma was the most frequently co-occurring keyword, appearing 166 times, highlighting its key role in the field.

**TABLE 4:** Most frequently Co-occurring keywords in ocular tumor-related metabolite research.

Index	Keyword	Frequency	Link strength
1	uveal melanoma	166	1,199
2	expression	157	1,161
3	cancer	132	931
4	retinoblastoma	117	806
5	biomarkers	116	861
6	prognosis	71	536
7	survival	71	533
8	metastasis	56	486
9	proliferation	49	366
10	breast-cancer	47	326
11	cells	47	336
12	retinoblastoma protein	47	287
13	gene-expression	46	285
14	mutations	45	327
15	progression	41	341

Further analysis of the co-occurrence network (Figures 3B–D) reveals several prominent research trends within ocular tumor-related metabolite studies. These include the exploration of immunotherapy approaches, the identification and validation of biomarkers, the investigation of tumor metastasis mechanisms, gene expression and mutation analyses, and the role of metabolites in ocular tumor development. These areas of focus are crucial for advancing our understanding of ocular tumors and laying the groundwork for the development of innovative diagnostic and therapeutic strategies.

### Keyword clustering and temporal evolution analysis

A keyword clustering analysis was conducted using the CiteSpace visualization tool, supplemented with the Log-Likelihood Ratio (LLR) algorithm, to identify major research themes within the field of ocular tumor-associated metabolomics (Figure 3F). This approach groups frequently co-occurring keywords into distinct clusters, representing cohesive and thematically relevant subfields. The clustering results, summarized in Table 5, revealed 11 prominent keyword clusters, each representing a key research direction. The modularity Q value was 0.76, and the silhouette S value was 0.8633—both well above standard thresholds (Q > 0.3 and S > 0.5)—indicating strong clustering quality and internal consistency. These values confirm that the clustering results are statistically meaningful and reflect reliable topic groupings within the literature.

**TABLE 5:** Keyword clustering analysis of research themes in ocular tumor metabolomics.

Index	Cluster label	Cluster size	Silhouette value
1	#0 circulating	41	0.912
2	#1 cells	40	0.871
3	#2 cell cycle	33	0.856
4	#3 prostate cancer	27	0.821
5	#4 cell lung cancer	25	0.772
6	#5 melanoma	25	0.821
7	#6 breast cancer	24	0.944
8	#7 circulating tumor dna	23	0.839
9	#8 retinoblastoma protein	22	0.791
10	#9 palbociclib	19	0.858
11	#10 urothelial carcinoma	19	0.891

To analyze how research themes have evolved over time, we employed a cluster peak diagram and timeline visualization based on the clustering output (Figures 3E,G). The cluster peak diagram highlights the time periods when each cluster was most active, while the timeline view provides a chronological map of keyword emergence and persistence. These tools jointly reveal both the temporal distribution and thematic trajectory of research developments from 2000 to 2024.

Our results demonstrate a clear temporal shift in research priorities. Before 2010, the focus was primarily on foundational cancer-related topics, such as “cell cycle,” “retinoblastoma protein,” “cancer,” “uveal melanoma,” and “survival,” indicating a strong emphasis on clinical prognosis and histopathological characterization. After 2011, the field transitioned toward more mechanistic and molecular-level investigations, with keywords such as “gene,” “mutations,” “resistance,” and “ribociclib” gaining prominence. This reflects a shift toward targeted therapy development, molecular diagnostics, and precision oncology in ocular tumor research.

Overall, the keyword clustering and timeline analysis reveal a progressive evolution of research from descriptive and clinical themes toward metabolic reprogramming, multi-omics integration, and therapeutic biomarker discovery. These trends are in line with global efforts to apply high-throughput metabolomics technologies to uncover novel diagnostic markers and therapeutic targets in ocular oncology. The incorporation of these analytical tools allows for an intuitive understanding of how the field has developed over time and offers guidance for identifying underexplored areas and emerging hotspots.

### Keyword burst analysis

**FIGURE 4:**
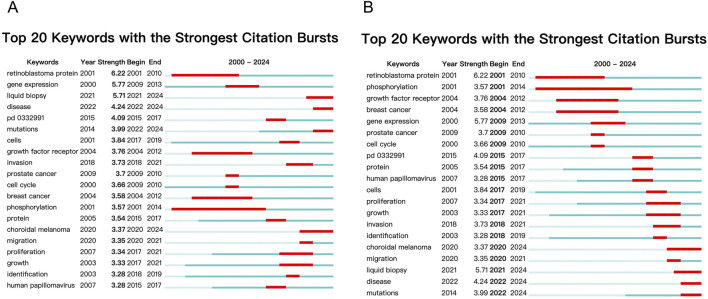
Keyword Burst Analysis. **(A)** Burst detection analysis showing keywords with significant shifts in frequency over time. **(B)** Temporal shifts in research hotspots.

A keyword burst analysis was conducted using the burst detection function in CiteSpace to identify keywords that have experienced significant shifts in frequency over short periods, providing insights into past and current research trends (Figures 4A,B). The analysis of burst intensity revealed that retinoblastoma protein (6.22), gene expression (5.77), liquid biopsy (5.71), and disease (4.24) exhibited the highest burst intensities in the periods 2001-2010 and 2009-2013. Notably, retinoblastoma protein and gene expression were the most prominent research areas during these periods.

From a temporal perspective, prior to 2010, research was predominantly focused on topics such as retinoblastoma protein, phosphorylation, growth factor receptors, breast cancer, gene expression, prostate cancer, and the cell cycle. Among these, retinoblastoma protein displayed a particularly high burst intensity, indicating its significant role in the research landscape of ocular tumor-related metabolites. From 2010 to 2020, emerging research hotspots shifted towards areas such as protein, human papillomavirus, cell proliferation, growth, and invasion. These topics dominated the research discourse during this decade, highlighting the growing focus on cellular and molecular mechanisms in ocular tumor-related studies. Currently, ongoing research continues to emphasize fields like choroidal malignant melanoma, liquid biopsy, and disease mutations, suggesting that these areas are likely to remain central research hotspots in the coming years. The sustained interest in these themes points to their continuing relevance and potential for future breakthroughs in the study of ocular tumors.

### Co-citation analysis

**FIGURE 5:**
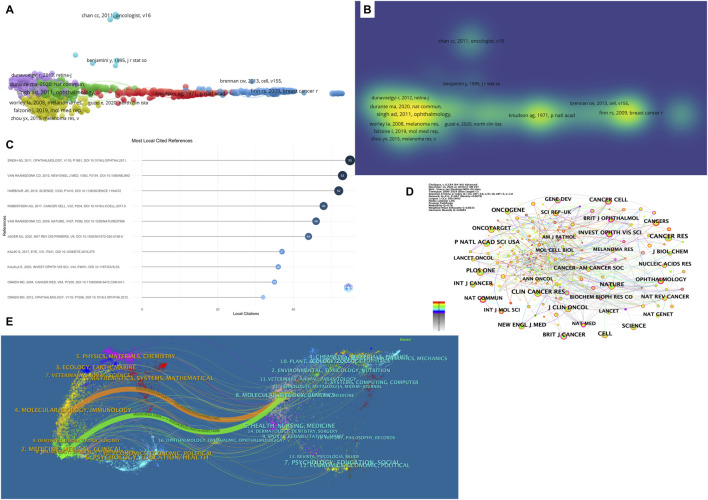
Co-Citation and Journal Analysis in Ocular Tumor-Related Metabolite Research. **(A)** Co-citation network map visualized with VOSviewer, displaying clusters of highly co-cited references. The size of each node reflects the number of citations. **(B)** Density visualization of co-cited references. **(C)** Most local cited references displayed as a bar chart. **(D)** Co-cited journal network visualization, highlighting journals with significant contributions to ocular tumor metabolomics. **(E)** Dual-map overlay of journals, illustrating citation flow between citing journals (left) and cited journals (right). Key pathways connect clinical and molecular biology research.

Co-citation analysis enables the identification of key research articles that are frequently cited together, providing insights into the foundational literature and current hotspots within a specific research domain. Table 6 presents the top 10 most co-cited articles in the field of ocular tumor-related metabolites, which serve as essential theoretical foundations for understanding key developments in this area. Figures 5A,B present the co-citation analysis and density visualization, while Figure 5C showcases the Most Local Cited References. The most frequently cited article, with 55 citations, is by Singh AD, published in 2011 in Ophthalmology under the title Uveal melanoma: trends in incidence, treatment, and survival. This paper provides valuable long-term data on the incidence, treatment, and survival trends of uveal melanoma in the United States. It highlights the lack of significant survival improvements despite changes in treatment approaches over time. The second most cited paper, with 53 citations, is authored by van Raamsdonk CD, published in 2010 in The New England Journal of Medicine under the title Mutations in GNA11 in Uveal Melanoma. This study offers an in-depth analysis of the role of GNA11 mutations in uveal melanoma, emphasizing the connection between these mutations and disease prognosis as well as the tumor’s biological characteristics. While these two papers focus on different aspects of uveal melanoma, they both provide crucial insights into the understanding and treatment of the disease. Their findings are interrelated, particularly regarding treatment trends and survival outcomes, forming a significant theoretical basis for ongoing research in the field.

**TABLE 6:** Top 10 most Co-cited references in ocular tumor metabolomics research.

Index	Citation frequency	Link strength	Cited references
1	55	1,525	singh ad, 2011, ophthalmology, v118, p1881, doi 10.1016/j.ophtha.2011.01.040
2	53	1,707	van raamsdonk cd, 2010, new engl j med, v363, p2191, doi 10.1056/nejmoa1000584
3	52	1,827	harbour jw, 2010, science, v330, p1410, doi 10.1126/science.1194472
4	48	1,325	robertson ag, 2017, cancer cell, v32, p204, doi 10.1016/j.ccell.2017.07.003
5	46	1,500	van raamsdonk cd, 2009, nature, v457, p599, doi 10.1038/nature07586
6	44	975	jager mj, 2020, nat rev dis primers, v6, doi 10.1038/s41572-020-0158-0
7	37	1,236	kaliki s, 2017, eye, v31, p241, doi 10.1038/eye.2016.275
8	36	875	kujala e, 2003, invest ophth vis sci, v44, p4651, doi 10.1167/iovs.03-0538
9	35	1,279	onken md, 2004, cancer res, v64, p7205, doi 10.1158/0008-5472.can-04-1750
10	32	1,188	onken md, 2012, ophthalmology, v119, p1596, doi 10.1016/j.ophtha.2012.02.017

### Journal visualization analysis

Table 7 lists the top ten journals publishing research in the field of ocular tumor-related metabolites, helping to delineate the thematic distribution of studies in this area. Among these, journals with over 300 published articles include Cancer Research (409 articles), Clinical Cancer Research (347 articles), Nature (341 articles), Proceedings of the National Academy of Sciences United States (317 articles), and Cell (300 articles). Figure 5D presents a visualization of co-cited journals.

**TABLE 7:** Leading journals publishing on ocular tumor-related metabolite research.

Index	Publication volume	Betweenness centrality	Year	Journal name
1	409	0	2000	CANCER RES
2	347	0.01	2002	CLIN CANCER RES
3	341	0.04	2000	NATURE
4	317	0.08	2000	P NATL ACAD SCI USA
5	300	0.03	2000	CELL
6	290	0.01	2001	ONCOGENE
7	285	0.05	2012	PLOS ONE
8	262	0	2000	SCIENCE
9	256	0.02	2003	J CLIN ONCOL
10	249	0.15	2001	BRIT J CANCER

The dual-map overlay of journals reveals key trends, such as the distribution of papers, citation trajectories, and the shift in research focus over time (Figure 5E). In this dual-map overlay, the left side represents the citing journals, while the right side represents the cited journals. The left-side labels indicate the academic fields of the cited journals, and the right-side labels indicate the fields of the cited papers. Most of the publications are found in journals related to molecular biology, while the majority of the citations appear in journals related to molecular biology, immunology, and medicine. This suggests that these journals are at the forefront of research in ocular tumor-related metabolites, reflecting the high level of interest in this subject area.

## Discussion

This study presents a comprehensive bibliometric analysis of global research on ocular tumor-associated metabolites, offering valuable insights into the developmental trajectory, thematic evolution, and collaborative dynamics in the field. Through the analysis of publication trends, institutional and national contributions, author networks, and keyword co-occurrence, we identified key research foci—such as uveal melanoma, retinoblastoma, metabolic biomarkers, and immunotherapy—as well as emerging directions like liquid biopsy and gene-metabolite interactions. Geographically, the United States and China have emerged as leading contributors, with a clear trend toward increasing participation from institutions worldwide. Thematic analyses reveal a shift from generalized molecular profiling toward a deeper exploration of metabolic reprogramming and its implications for tumor progression and treatment. Co-citation networks and high-burst keywords also highlight the field’s foundational literature and emerging scientific attention. Overall, our findings provide an updated structural overview of the ocular metabolomics research landscape and highlight future directions for advancing precision diagnostics and metabolite-based therapies in ocular oncology.

Despite the growing volume of research output—particularly since 2016—our analysis also uncovered a notable decline in citation frequency beginning around 2019, indicating a gap between research productivity and scholarly impact. Several factors may account for this phenomenon. First, a citation lag is expected for more recent publications, which have not yet had sufficient time to accumulate influence. Second, the expansion of research output may result in diluted citation attention, reducing the average number of citations per study. Third, a proportion of newer articles may be descriptive or lack strong methodological innovation, leading to limited visibility or clinical relevance. Additionally, some studies may suffer from limited dissemination due to weak international collaboration networks or publication in lower-impact journals. Together, these factors suggest that future efforts in ocular tumor metabolomics should emphasize not only quantitative growth, but also scientific rigor, translational depth, and strategic collaboration to enhance global visibility and impact.

The geographical distribution of research contributions, with the United States maintaining its leadership and China rapidly increasing its output, underscores the growing global interest in ocular metabolomics. However, the observed fragmentation in institutional collaboration, despite the prominence of top institutions in the U.S. and China, reveals a critical opportunity for strengthening cross-border partnerships. Collaborative networks could accelerate the pace of discovery by fostering diverse perspectives and sharing research resources, especially in a field that increasingly relies on complex technologies and interdisciplinary approaches. Enhanced international cooperation may be essential for overcoming current challenges and driving the field towards more impactful outcomes.

The evolving focus of research, as reflected in keyword trends, signals a shift toward deeper exploration of the molecular and genetic mechanisms underlying ocular tumors, particularly uveal melanoma. The prominence of keywords such as “liquid biopsy,” “retinoblastoma protein,” and “gene expression” reflects a growing emphasis on precision medicine, where understanding the genetic basis of ocular tumors is central to developing personalized diagnostic and therapeutic strategies (Yu et al., 2022; Engeland, 2022; Bian et al., 2022). This trend aligns with broader advancements in cancer metabolomics and biomarkers, highlighting the need for continued innovation in non-invasive diagnostic techniques and targeted therapies. As the field matures, the integration of metabolomic profiles with genetic and clinical data will likely enable more effective identification of biomarkers for early detection, prognosis, and treatment response, ultimately leading to improved patient outcomes.

With the rapid advancements in high-throughput metabolomics technologies, ocular tumor metabolism research has made significant strides, unveiling previously unexplored aspects of tumor biology. Techniques such as liquid chromatography-tandem mass spectrometry (LC-MS/MS), gas chromatography-mass spectrometry (GC-MS), and nuclear magnetic resonance (NMR) have emerged as powerful tools for comprehensive analysis of ocular tumor metabolic profiles. These methods enable the quantification and identification of a broad spectrum of metabolites, including amino acids, fatty acids, glucose, and lipids, providing insights into the metabolic reprogramming that occurs during tumorigenesis. For example, LC-MS/MS has been employed to analyze serum, urine, and tissue samples from patients with retinoblastoma and uveal melanoma, revealing significant alterations in metabolites such as omega-3 and omega-6 fatty acids, glutamate, and alanine, which are closely associated with tumor proliferation, invasion, and metastatic potential (Luo et al., 2019; Khade et al., 2024). Similarly, GC-MS has been used to identify volatile and semi-volatile metabolites linked to lipid metabolism and oxidative stress, which are key contributors to tumor malignancy and chemoresistance (Pattarachotanant and Tencomnao, 2020). NMR spectroscopy, with its non-destructive nature, offers a promising approach for *in vivo* monitoring of metabolic changes, and has been successfully used to detect unique metabolic signatures in the blood and urine of retinoblastoma patients (Gulati et al., 2024). Moreover, the integration of metabolomics with systems biology approaches, such as metabolic network modeling and transcriptomic data integration, has provided new frameworks for understanding the intricate metabolic pathways that support ocular tumor progression. These advances have led to the identification of critical metabolic pathways, including lipid metabolism, amino acid metabolism, and glycolysis, all of which are reprogrammed in ocular tumors to support cell growth, survival, and adaptation to the tumor microenvironment (Konaklieva and Plotkin, 2024).

Despite the significant advancements in metabolomics techniques in the field of ocular tumor research, several challenges remain. One of the primary difficulties lies in the distinct metabolic characteristics of ocular tumors compared to other types of cancers (Schrauwen-Hinderling and Schols, 2018). These differences are attributed to the unique microenvironment of the eye, including factors such as the limited blood supply, local immune responses, and the ocular tissue’s inherent metabolic properties (Gottfried et al., 2012). The complexity of the ocular tumor microenvironment presents significant obstacles in accurately identifying and characterizing tumor-specific metabolites, which in turn complicates the development of reliable biomarkers for early detection and prognosis (Zhang et al., 2022). Currently, most studies focusing on ocular tumor-related metabolites tend to concentrate on a limited number of classical metabolic pathways and specific metabolites, such as those related to glucose metabolism, lipid metabolism, and amino acid signaling. However, large-scale, comprehensive metabolomics studies that investigate the entire metabolic network of ocular tumors are still lacking. Furthermore, there is a lack of cross-disciplinary research that integrates metabolomics with genomics, proteomics, and other omics technologies, which limits our ability to fully understand the intricate mechanisms of metabolic reprogramming in ocular tumors.

In addition to the identification of altered metabolites, recent studies have shed light on the molecular mechanisms and metabolic-related targets that drive ocular tumor progression. For instance, in uveal melanoma, enzymes involved in fatty acid synthesis, such as Fatty Acid Synthase (FASN), and in fatty acid oxidation, like Acyl-CoA Dehydrogenase (ACAD), are upregulated, supporting enhanced lipid metabolism and providing energy to the rapidly proliferating tumor cells (Han et al., 2023; Ma et al., 2021). Hexokinase 2, a key enzyme in the glycolytic pathway, is also significantly overexpressed in both retinoblastoma and uveal melanoma, facilitating the shift to aerobic glycolysis, a hallmark of cancer cell metabolism (Tang et al., 2021; Liu et al., 2018). Transcription factors, particularly hypoxia-inducible factor 1-alpha (HIF-1α), also play a central role in metabolic reprogramming by activating genes involved in glycolysis and lactate fermentation under low oxygen conditions, contributing to tumor aggressiveness and drug resistance (D'Aguanno et al., 2021). The altered metabolic microenvironment in ocular tumors, marked by increased lactate and ammonia production, also promotes immune evasion by lowering the local pH, thus hindering the antitumor immune response. As a result, lactate metabolism has emerged as a potential therapeutic target, with studies suggesting that targeting lactate dehydrogenase (LDH) could inhibit tumor progression and enhance the efficacy of immunotherapy (Longhitano et al., 2022). Furthermore, specific metabolites, such as lactate, glutamate, and triglycerides, have been identified as promising biomarkers for the early diagnosis and staging of ocular tumors, providing a foundation for the development of non-invasive diagnostic tools (Nathan et al., 2021; Tonelotto et al., 2024). Collectively, these findings highlight the growing importance of metabolic pathways in ocular tumor biology, emphasizing the need for targeted therapeutic strategies that modulate tumor metabolism and microenvironment to improve patient outcomes.

As ocular tumor metabolomics continues to evolve, several promising research directions are emerging. First, there is a clear need to deepen our understanding of specific metabolites that could serve as biomarkers for early detection of ocular tumors, particularly in uveal melanoma and retinoblastoma. Liquid biopsy, as indicated by recent keyword trends, presents an exciting avenue for non-invasive detection, and future studies should focus on identifying metabolites that significantly change during the early stages of these malignancies. As liquid biopsy emerges as a promising non-invasive approach to accessing ocular tumor biology, its clinical translation warrants a structured implementation framework. First, it is essential to critically evaluate the clinical applicability of metabolite biomarkers identified in preclinical studies, assessing their diagnostic, prognostic, and predictive value in real-world patient cohorts. Second, the field would benefit from the establishment of a curated, multi-center ocular tumor metabolite database, cataloging aqueous humor and vitreous biomarkers across tumor types, stages, and treatment responses. Third, the development of standardized protocols for sample collection—such as anterior chamber paracentesis—and metabolomic profiling will be crucial to ensure reproducibility and data harmonization. Finally, clinical trial designs based on metabolic subtypes (e.g., glycolytic vs. lipid-driven tumors) could enable personalized treatment regimens and biomarker-guided therapeutic monitoring. Together, these efforts would greatly enhance the translational value of metabolomic research in ocular oncology and facilitate its integration into precision medicine pathways.

Secondly, the intricate relationship between metabolic pathways and gene expression warrants further exploration. Integrating metabolomics with genomic data will provide valuable insights into the molecular mechanisms driving ocular tumorigenesis, with particular emphasis on how metabolic reprogramming influences gene expression in ocular tumor cells. Moreover, the role of the tumor microenvironment in influencing metabolic reprogramming is a critical area that deserves greater attention. As ocular tumors such as uveal melanoma exhibit distinct metabolic signatures, understanding how these changes interact with immune cells and other components of the microenvironment could unlock new therapeutic strategies. In addition, personalized treatment approaches based on metabolomic profiling are likely to revolutionize ocular tumor management (Demirci et al., 2019). By tailoring therapies to individual metabolic signatures, clinicians can optimize treatment efficacy and minimize adverse effects (Márquez and Matés, 2021). Finally, advancing metabolomic analysis technologies remains essential for the continued progress of this field. Innovations in high-throughput platforms, such as mass spectrometry and nuclear magnetic resonance, will allow for more precise identification and quantification of metabolites, thereby facilitating the discovery of new biomarkers and improving diagnostic capabilities. In summary, these research directions offer considerable promise for enhancing our understanding of ocular tumor biology and advancing personalized medicine in this field.

While this bibliometric analysis provides valuable insights into the trends and developments in ocular tumor metabolomics, several limitations should be acknowledged. First, this study relied solely on the Web of Science Core Collection (WoSCC), which, although selected for its comprehensive coverage and compatibility with bibliometric tools, may exclude certain publications indexed only in Scopus or PubMed. However, a supplementary comparison indicated that WoSCC includes the vast majority of relevant records from these databases, supporting the validity of our dataset and analysis. Future studies may benefit from integrating multiple databases to further enhance coverage, particularly for region-specific or emerging literature. Furthermore, although the growing number of publications in ocular tumor metabolomics is encouraging, this surge in quantity does not necessarily correlate with an improvement in quality. The methodological rigor and clinical relevance of these studies can vary significantly, with some research lacking robust experimental designs or real-world applicability. As a result, it is crucial to consider not only the sheer volume of publications but also their scientific and clinical contributions. Additionally, citation practices, which differ across countries and institutions, may introduce biases that skew citation trends. For example, articles from certain regions may receive disproportionately high citations due to local academic networks, while impactful work from underrepresented areas could be overlooked. To gain a more accurate understanding of the field’s development, future analyses could explore citation networks and impact factors to provide a more nuanced interpretation of research impact. Despite these challenges, the insights provided by this analysis contribute to a broader understanding of the field’s trajectory, while also highlighting areas where future research and methodological improvements are needed.

## Conclusion

This bibliometric analysis highlights the significant strides made in ocular tumor metabolomics research, underscoring both the increasing volume of publications and the growing geographical and institutional diversity of contributions. Despite the surge in research activity, challenges remain in enhancing the impact of these studies, fostering greater international collaboration, and addressing gaps in translational applications. Moving forward, integrating novel technologies and refining collaborative networks will be crucial for advancing our understanding and improving the diagnosis and treatment of ocular tumors.

## Data Availability

The original contributions presented in the study are included in the article/Supplementary Material, further inquiries can be directed to the corresponding authors.
