# Supplementary figures and images for: Global trends and emerging insights in ocular tumor-associated metabolites: a bibliometric and visualization analysis
Source: Front Mol Biosci. 2025 Jun 18;12:1572710. doi: 10.3389/fmolb.2025.1572710 (PMC12213345; doi:10.3389/fmolb.2025.1572710)

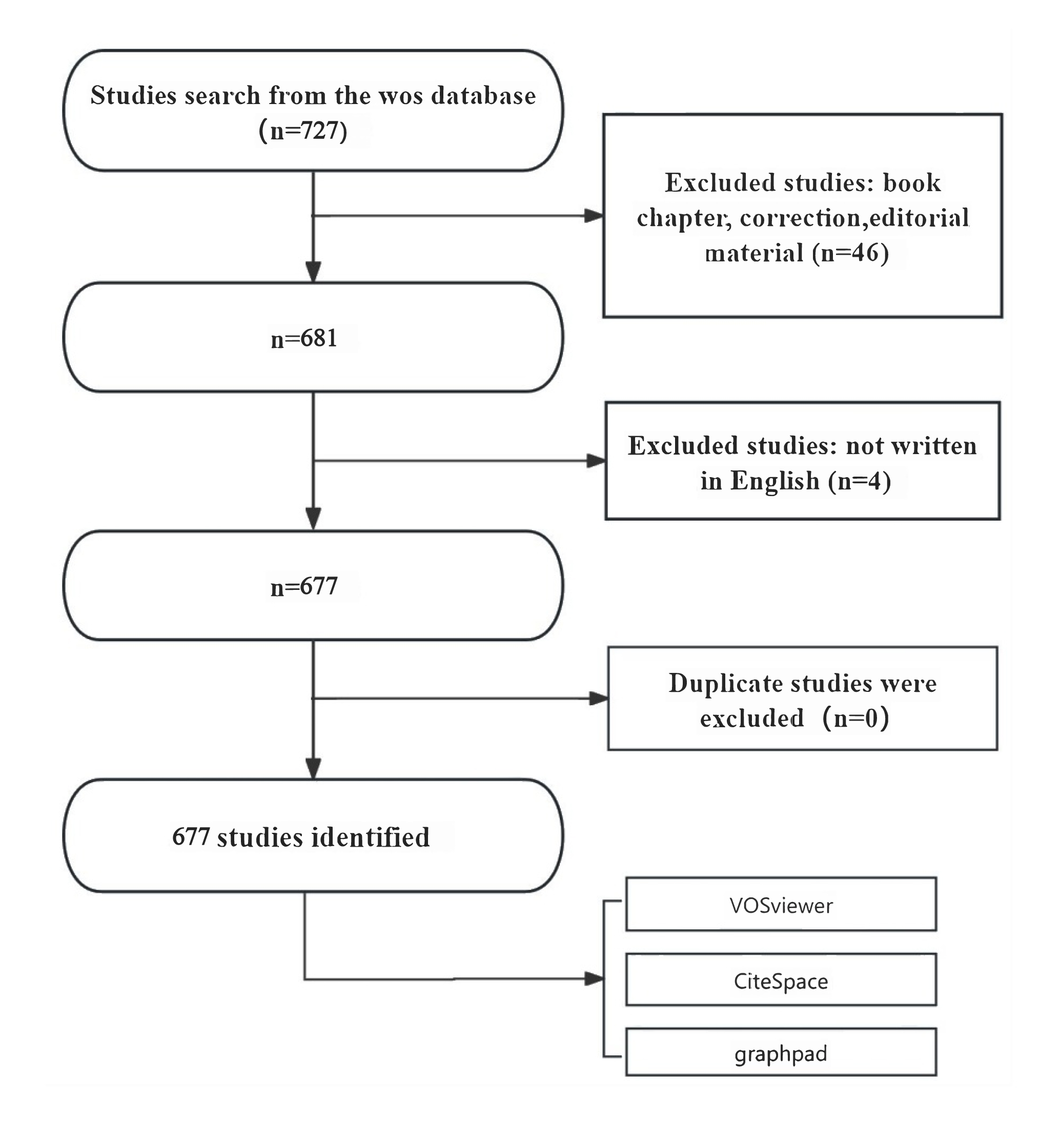

Supplement: Supplementary file 1 [file Image1.jpeg]
